# Sudden Cardiac Arrest in Athletes With Cardiomyopathies: Focusing on the Fundamentals

**DOI:** 10.1016/j.jacadv.2026.103249

**Published:** 2026-10-21

**Authors:** Eli M. Friedman, Matthew W. Martinez

**Affiliations:** a Department of Cardiology, Smidt Heart Institute, Cedars-Sinai Medical Center, Los Angeles, California, USA; b Department of Cardiology, Mayo Clinic, Jacksonville, Florida, USA

**Keywords:** arrhythmogenic cardiomyopathy, athlete, exercise, hypertrophic cardiomyopathy, sudden cardiac death

**Figure:**
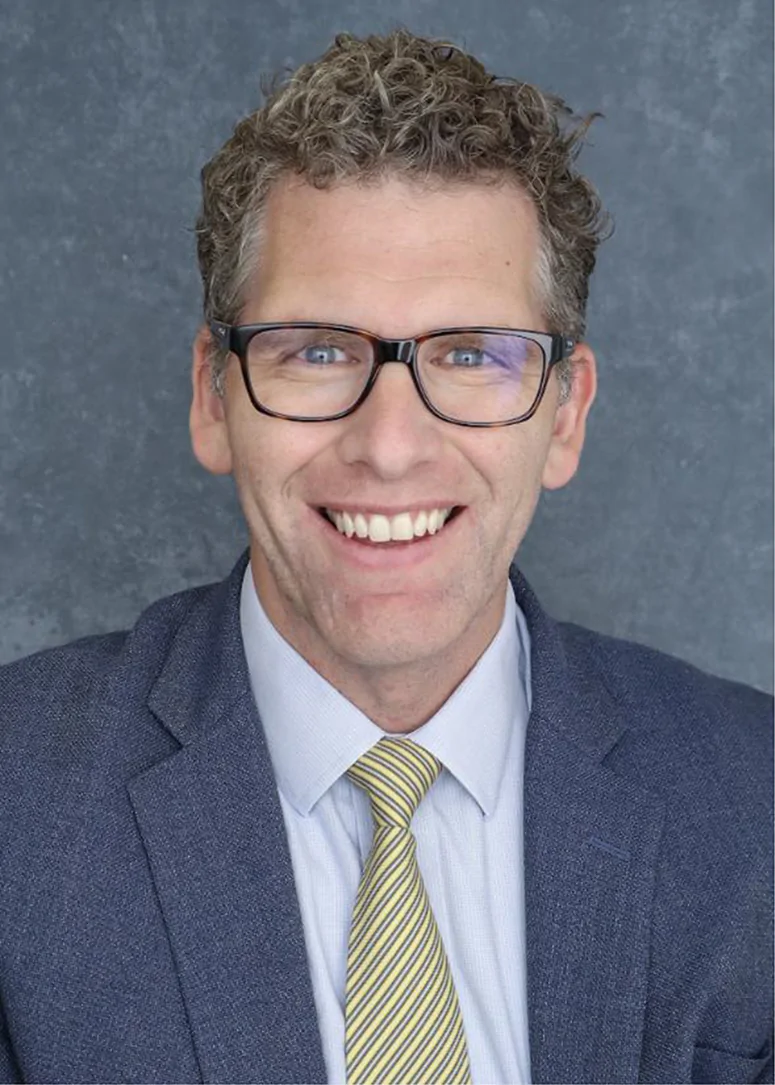

Sudden cardiac arrest (SCA) in a young athlete is a highly visible, emotionally charged event. The incidence of these events varies based on demographics. In adolescents, the incidence of SCA is estimated at 1 case in 51,000 to 88,000.^1^, ^2^, ^3^, ^4^ While the potential etiologies that cause SCA in young athletes are numerous, the most common ones are autopsy-negative sudden unexplained death, hypertrophic cardiomyopathy (HCM), and idiopathic left ventricular hypertrophy representing a possible cardiomyopathy.^4^

SCA in an athlete leads to public scrutiny with an increased focus and desire for advanced preparticipation cardiac screening to prevent the next event. However, the focus should be on the role of the emergency action plan (EAP) and prompt automated external defibrillator (AED) use, which are the most important ways to respond to SCA.

In this issue of *JACC*: *Advances*, Petek et al^5^ describe the demographics, circumstances, and resuscitative efforts for athletes with SCA related specifically to cardiomyopathies. Using the University of Washington National Collegiate Athletic Association Athlete Death Database and the National Center for Catastrophic Sports Injury Research Database, 454 deaths were identified. Of these deaths, 129 were attributed to cardiomyopathies in athletes aged 11 to 27 years. Eighty-one were attributed to HCM, representing 62.8% of the cohort. HCM was the most common cardiomyopathy identified. Arrhythmogenic cardiomyopathy was the second most common etiology of SCA with an incidence of 22 (17.1%) and 26 were classified as other cardiomyopathies (20.2%). The majority of the events occurred at a sporting facility (87/129, 67.4%), with most occurring during training or practice (74.7%) as opposed to formal competition (25.3%). Survival rate was 34.9% and survivors were more likely to receive bystander cardiopulmonary resuscitation (CPR; 97.6%) and have an AED (72.4%) use on them. Coaches were the most frequent first responders (40%), followed by athletic trainers (35%), and nurses (15%). The study does not tell us how many of these cardiomyopathies had been diagnosed previously and the status of EAPs/cardiac emergency response training of those who responded to these athletes.

The authors of this study do an excellent job of highlighting and amalgamating two important, complimentary areas of consideration for anyone who is a stakeholder in the health and safety of athletes. First, although HCM is no longer considered the single most common etiology of SCA in athletes, underlying cardiomyopathies continue to be a frequent diagnosis associated with cardiac emergencies in sports.^4^^,^^6^ While this may reignite the debate regarding enhanced preparticipation cardiac screening in athletes with an electrocardiogram and/or echocardiogram, modern data tell us that regardless of screening or a known diagnosis of cardiomyopathy, SCA may still occur.^2^ However, screening to find a diagnosis of an athlete with a cardiomyopathy or any etiology associated with SCA does allow for sport and position-specific risk stratification and development of a plan for athletes to participate in high-intensity, high-volume activity with expert sports cardiology oversight and guidance on the necessity of EAPs.^7^ Thus, more athletes with cardiomyopathies are likely to engage in high-intensity, high-volume exercise.

This brings us to the second and perhaps the most important part of the study: the timing of when SCA occurs, the response to SCA, and who the first responders are. Most of the SCA events happened during periods of practice or training rather than during competition, and coaches were the most frequent first responders. Some of the most recent highly publicized cardiac emergencies in sports occurred in athletes who had undergone and cleared intensive cardiac screening. It was the execution of well-rehearsed EAPs leading to timely recognition, response, and treatment of SCA which ultimately saved the lives of those athletes. This reminds us that even when preparticipation cardiac screening is executed to perfection, cardiac emergencies will still happen. Despite the knowledge that EAPs are critical to athletes’ health and safety, opportunities abound to improve emergency response and expand the pool of first responders. In the United States, 30% of youth coaches received education and training on cardiac emergencies in 2024 despite this being a top priority for parents.^8^ A study in Cameroon showed that only 12% of stakeholders in soccer had confidence in CPR or AED use, and large gaps existed in EAPs, CPR training, and AED accessibility.^9^

Progress is being made, though. The Smart Heart Sports Coalition, led by the National Football League with contributions from other major sports organizations, has led advocacy efforts for all 50 states to adopt legislation for CPR and AED training for all coaches, clearly marked AEDs within 3 minutes of venues that sponsor athletic activity, and EAPs for those same venues. Calls have been made to widen the pool of first responders to cardiac emergencies,^10^ and organizations such as the Women’s Tennis Association and Major League Soccer have followed suit by training chair umpires and on-field officials, respectively. This represents a paradigm shift of leading by example, demonstrating preparedness at the highest levels of sport openly, and inspiring grass roots action in communities.

Petek et al^5^ remind us that expanding the pool of those trained to respond to cardiac emergencies should be considered for anyone aiming to reduce adverse cardiac events in and around sports. Despite best efforts with screening, risk stratification, and treatment, cardiac emergencies in sports relating to cardiomyopathies will continue to occur. The inclination will always be to ask after the event, “Why did this happen?” However, we encourage all stakeholders to ask *before* the next event happens if they know how to recognize a cardiac emergency, if they would be comfortable responding to it, and if their organization’s EAP has been rehearsed within the last 6 to 12 months (if it exists at all)? Answers to these questions lead to action in preparing for emergencies, which ultimately is the keystone for athletes’ health and safety.

## Funding support and author disclosures

Dr Friedman has received compensation from the Women’s Tennis Association and Ultimate Fighting Championship’s Combat Sports Anti-Doping Program as a consultant. Dr Martinez has received grant funding/consultancy compensation from the National Hockey League, National Basketball Players Association, and Major League Soccer.
